# A Variable-Impulse Hammer Impact Test (VIHIT) Method for Improved Mode Shape Identification

**DOI:** 10.3390/s26092712

**Published:** 2026-04-28

**Authors:** Alec Jensen, Charles Riley

**Affiliations:** Civil Engineering Department, Oregon Institute of Technology, Klamath Falls, OR 97601, USA; jensen.alecj@gmail.com

**Keywords:** impact hammer, impulse hammer, modal analysis, vibration, nonlinear structures, civil structural health monitoring, modal parameter identification

## Abstract

The impact hammer, equipped with a force transducer, is a portable and practical tool for inducing measurable excitations in structural health monitoring (SHM). However, its reliability is often limited by uncontrolled factors such as swing power, angle, impact location, and operator consistency, particularly in nonlinear structures operating at low frequencies. While many researchers have avoided hammer testing by instead using better controlled drop mass systems or operational modal analysis (OMA) techniques, this study presents a new experimental modal analysis (EMA) approach that improves the accuracy of impact hammer testing: variable impulse hammer impact testing (VIHIT) using a single-input single-output (SISO) roving hammer and single fixed accelerometer. For a mode of interest, the imaginary component of the frequency response function (FRF) is evaluated at each test location using multiple impulses of varying magnitude. This output quantity exhibits an inverse power relationship with the input autopower spectral density (APSD) at the modal frequency. Evaluating the trend at a reference input APSD from sufficiently excited tests produces a very accurate mode shape for that input. For a given structure, nonlinear damping ratios vary with excitation and can be extracted using inverse FRF analysis. This method addresses variability in impact hammer testing by establishing reproducible trends for different impulse levels and test locations. Application to degraded timber beams demonstrated reductions in mode shape variability relative to conventional averaging and revealed impulse-dependent damping ratios ranging from approximately 0.02 to 0.04, highlighting the method’s ability to characterize nonlinear dynamic behavior. The result is a more accurate approach for extracting modal properties and mode shapes and characterizing nonlinear dynamic behavior using a SISO roving impact hammer system.

## 1. Introduction

The impact hammer is an instrument designed to supply and measure impulse using a force transducer integrated into its head. Its primary use is in assessing the dynamic performance of structures. Larger hammers are suited to exciting full-scale civil structures such as bridges and buildings, while smaller models are commonly used in mechanical applications such as automotive components, machinery parts, precision instruments, or small-scale assemblies. Impact hammers are commonly used in applications such as non-destructive testing (NDT) for SHM, finite element model (FEM) validation, and vibration testing. When combined with an accelerometer that records the response of an impacted structure, hammer measurements enable the generation of a normalized transfer function known as the frequency response function (FRF). FRFs can be analyzed to extract the modal characteristics of the structure, including mode shapes and associated damping ratios and natural frequencies. However, in nonlinear structures with low natural frequencies, impact hammer testing often fails to capture accurate results because the structure’s response can vary with the magnitude of the impulse.

Impact hammers are criticized for inconsistent results due to a lack of control over factors like swing power, angle, location, choice of tip material, and operator consistency. The deterministic yet difficult-to-replicate nature of the impulse makes consistent outcomes difficult to achieve [1,2,3,4]. This is especially significant when test subject behavior is nonlinear, especially at lower modal frequencies [5]. Other researchers have used impact hammer control jigs or systems with controllable force (like drop mass systems); however, with large, hand-operated hammers meant for larger structures, this is not ideal and limits the portability of a hammer system. While many researchers focus on controlling the impulse, this paper introduces a new experimental modal analysis (EMA) method that applies multiple increasing impulse magnitudes to account for variations in response relative to impulse intensity. The method can more accurately assess low-frequency modes in nonlinear structures through experimental modal analysis (EMA) by using a very portable single-input single-output (SISO) roving hammer approach with a fixed accelerometer setup.

The remainder of this paper is organized as follows. Section 2, Literature Review, summarizes prior work relevant to this study. Section 3, Methods and Materials, outlines the equipment, test subjects, proposed method, and pertinent theory. Section 4, Results and Discussion, presents the findings and evaluates the method’s effectiveness. Section 5, What Can Go Wrong?, reviews common impact testing issues and mitigation strategies. Section 6, Future Recommendations, suggests areas for continued research, and Section 7, Conclusion, summarizes the key findings.

## 2. Literature Review

When testing a linear system, averaging test results at each impact location helps filter out noise and variability and can aid in identifying erroneous data [6,7,8]. Averaging can also be used to develop a valid coherence plot for impact testing [9]. However, Halvorsen and Brown [6] explain that in nonlinear systems, the FRF is not uniquely defined and varies with the amplitude of the input excitation; therefore, averaging test results will not produce conclusive results and can depend on the range of impulses used. Hammer impacts can alter the structural response by exciting the structure outside of its linear range, causing geometric nonlinearities such as spring hardening and large deformations, and potentially exposing material nonlinearities, including stiffness softening caused by prior damage [5,10,11,12]. In standard impact testing, structural response that varies across a typical range of impact magnitudes can indicate the presence of nonlinear behavior in the dynamic response. This paper identifies the inverse power relationship of structural response to hammer impulse and uses this fact to improve the accuracy of modal parameter identification from SISO impact hammer testing.

More recent papers regarding impact hammers have transitioned to controlling the strikes with force-control systems [3,12,13]. Some examples of controlled impact include a specialized impact hammer used by Zheng et al. [5] to generate consistent and controllable impulses, and the Scalable Automatic Modal (SAM) hammer developed by Blaschke et al. [12], a small-scale motorized hammer mount designed for controlled impacts on lightweight structures. Both approaches enable the characterization of nonlinear behavior through controlled impulse magnitude. However, the solution presented in this paper uses a 5.5 kg hammer as-is, swung by a human operator, without any modifications or control systems. Given the hammer’s size, implementing a controlled impact mechanism would be challenging without cumbersome specialized equipment.

Lower frequency modes, associated with smaller eigenvalues and larger, global deformations, are more influenced by overall structural stiffness and typically involve greater mass participation, making them dominant in the system’s dynamic response. In contrast, higher frequency modes exhibit more localized deformation patterns and are therefore more sensitive to local variations in mass and stiffness [14]. Impulse loading predominantly excites lower frequency modes as demonstrated by Zheng et al. [5] and the results of this paper, as the input autopower spectral density (APSD) “rolls off” with increasing frequency, which in nonlinear structures, are more sensitive to geometric nonlinearities and stress softening or stiffening. Fladung [2] mentions that impacts are not generally a good excitation method for nonlinear structures due to the lack of control. However, this study successfully used an impact hammer to test low-frequency nonlinear systems without additional tooling or equipment.

One goal of this study is to support structural damage detection by analyzing location-specific dynamic responses. Modal analysis is particularly useful in this context as mode shapes can reveal localized changes in structural behavior caused by damage or structural property changes. Previous work has applied dynamic testing to detect damage by comparing responses of damaged and undamaged structures [15,16]. Other studies have relied solely on natural frequencies from impact hammer tests to assess structural integrity [17,18], though results can vary depending on factors such as mass loss and structural nonlinearity.

The signal-to-noise ratio (SNR) of impact hammers is often worse than other methods like shakers [19,20], making them unreliable and harder to interpret at times. EMA with impact hammers has many formats for displaying results such as grouping inverse FRFs to determine dynamic mass, dynamic stiffness, and damping coefficients [5,21] or extracting mode shapes, damping, or material properties from FRFs [8,15]. Setting aside other concerns, most research favors operational modal analysis (OMA) [22]. OMA is often preferred over EMA when conventional excitation methods are impractical due to the size of the structure, or when accessibility and real-time data collection are critical. However, OMA typically relies on ambient or routine loading, which may not sufficiently excite the structure to reveal its full dynamic behavior under larger loads. This limitation can lead to an incomplete or non-representative identification of modal properties such as natural frequencies and damping ratios, particularly those associated with higher energy or nonlinear response [23]. To explore these nonlinear effects in low-frequency systems, this study employed a straightforward EMA approach using a roving input SISO system.

Halvorsen and Brown [6] identify three types of nonlinearities that can occur in structural systems under impulse excitation: nonlinear damping, load-sensitive stiffness nonlinearity, and part clearance nonlinearity. Nonlinear damping often originates at structural joints, where energy dissipation depends on the relative displacement across the joint, leading to amplitude-dependent damping behavior. If the excitation is near a nonlinear damping site, increased relative motion causes the measured frequency response to show higher apparent damping. Load-sensitive stiffness, where the effective spring rate changes with the applied load, is a common structural nonlinearity that can either stiffen or soften the system response. A frequently encountered structural nonlinearity arises from part clearances between components, which can lead to inaccurate static stiffness estimates, poor repeatability in FRF measurements, and an overestimation of damping when using impulse excitation. Accordingly, the analysis in this paper accounts for these nonlinearities in its interpretation.

## 3. Methods and Materials

### 3.1. Equipment

**Figure 1:**
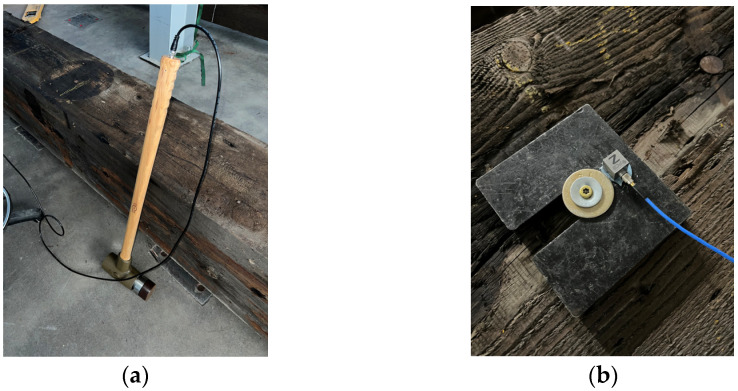
Setup photos of (**a**) PCB Piezotronics Model 086D50 Impulse Force Hammer equipped with the soft brown tip; (**b**) PCB Piezotronics Model 356A32 accelerometer attached with adhesive onto a steel plate secured to the test subject by a screw.

The hammer used in this research was the PCB Piezotronics (Depew, NY, USA) Model 086D50 Impulse Force Hammer equipped with the soft brown tip (Figure 1a). Tip hardness influences both contact duration and impact force, with harder tips resulting in shorter contact times and higher force amplitudes, generating broader high-frequency excitation. The softer tip was selected to better excite the lower frequency response of the heavy timber specimens. Tip choice entails another trade-off: because increased contact duration reduces high-frequency content, the excitation mechanism of nonlinear behavior may be altered as well. While this trade-off is recognized, its implications were not further explored in this testing.

The hammer has a sensitivity of 0.227 mV/N which was verified in a calibration validation test. It is considerably larger than other models, with a mass of 5.5 kg, and is well suited for larger structures in civil engineering applications where a larger excitation is necessary.

The accelerometer used was the PCB Piezotronics Model 356A32 with a sensitivity of 102.6 mV/g, which can be seen in Figure 1b.

Throughout testing, the accelerometer provided consistent, reliable measurements, and the frequency content of the response appeared as expected, with no sudden flattening indicative of overload. In this experiment, the accelerometer was mounted exclusively at the center of the simply supported beam’s span. This location was deliberately chosen because it corresponds to the antinodes of flexural modes 1 and 3, where the response is most pronounced. However, it is not sensitive to mode 2, as a simply supported beam has a node at the center in this mode, resulting in minimal or zero response at that point. This decision was made to understand the effects of nonlinearity between a lower and higher mode of interest and to maximize consistency and signal quality for the target modes of interest.

A goal of the work was to develop a highly portable system to facilitate field studies. Thus, the hammer and accelerometer were connected to a two-channel PCB Piezotronics 485B39 Signal Conditioner, which interfaced with an Apple (Cupertino, CA, USA) iPhone 13 Pro running the Vibration Pro app version 4.14 [24]. Signal processing and analysis were performed in GNU Octave version 10.1.0.

### 3.2. Timber Stringer Specimens and Concrete Bridge

**Figure 2:**
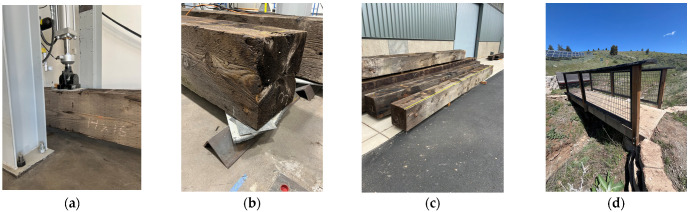
Photos of (**a**) timber in static loading with a string pot to measure displacement; (**b**) shimmed support; (**c**) timber stockpile; (**d**) concrete bridge.

The testing for this experiment utilized three Douglas fir timber bent caps removed from a bridge repair project in Klamath County, OR (Figure 2a), and a concrete bridge on the Oregon Institute of Technology, Klamath Falls campus (Figure 2b). The timbers had varying degrees of damage and wood-decay fungus from over 60 years of service and exposure. The timbers were simply supported with minimal overhang. Due to uneven support conditions caused by factors such as protruding original hardware and damaged or non-planar timber, shims were inserted between the beam and vertical supports, as shown in Figure 2c, to ensure good bearing contact, mitigate rocking, and prevent undesired torsional motion under excitation and static loading (Figure 2d). It is acknowledged that the shimmed support conditions may introduce boundary-related nonlinearities that are not isolated from material nonlinearity in this study. The proposed VIHIT methodology intentionally characterizes the combined effect of material degradation and support behavior through excitation-conditioned modal properties, rather than attempting to decompose individual nonlinear contributions.

The timbers were nearly 5.5 m in length but spanned 5.2 m between supports. The cross-sectional properties of the timbers are approximate, as their condition was affected by rot, damage, warping, and cracking, which introduced considerable uncertainty. The timbers were measured to have a width (*b*) of 343 mm and a depth (*d*) of 397 mm, resulting in an ideal moment of inertia (*I*) of 1.79 × $10^{9}\;{\mathrm{mm}}^{4}$. Since the timbers were degraded uniquely, there was no feasible way of developing the correct moment of inertia. Timbers 1, 2, and 3 had masses of approximately 577 kg, 513 kg, and 488 kg, respectively. Timber 1 initiated the removal of the bent caps from the bridge, as it was found to be significantly rotted, and of major concern to Klamath County engineers, but Timber 2 and 3 were determined to be more competent, based on resistograph testing. A significantly more competent reinforced concrete slab pedestrian bridge was also evaluated, providing a comparison to a system that behaved more consistently with linear predictions. The properties of the timbers and concrete bridge are reported in Table 1.

### 3.3. Proposed Experimental Method

The process for the proposed experimental method used in this study is as follows:

1. Impulse Testing and Data Acquisition

At each designated test location, 10 to 20 variable impulse tests are performed, slightly more than suggested for averaging methods [8,9], to ensure data quality and capture the full impulse range. To capture the full dynamic range of the structure’s response, impulses of varying magnitudes, from light taps (~10 N·s) to heavy strikes (~60 N·s), are applied at each location to ensure the input APSD spans a broad range and reveals both low and high impulse behavior. Consistency of the strikes across this range is impossible to control, given the test equipment, so the goal is to span the range to inform an impulse-dependent response, rather than reproduce a specific impulse value consistently.

2. Frequency Domain Processing

The recorded signals are transformed using Fast Fourier Transform (FFT). From this, the input APSD and the crosspower spectral density (CPSD) between input and output are calculated. The FRF is obtained by dividing the CPSD by the input APSD. More detail is provided in the *Signal Processing* sections that follow.

3. Imaginary FRF Trend Extraction

For each test at each location, the APSD of the input signal, $S_{xx}\left(f\right)$, is plotted as a function of the imaginary component of the FRF, $Im\left(H\left(f\right)\right)$, at the modal frequency of interest, ${f=\;f}_{n}$, where *n* is the mode number. The tests at each location collectively form a set of curves that typically follow an inverse power-law relationship of the form $Im\left(H\left(f_{n}\right)\right)=\;a\cdot {{[S}_{xx}\left(f_{n}\right)]}^{-b}$. The shape of these curves provides insight into the system’s impulse-dependent dynamics at the mode and location of interest. Additionally, this inverse power relationship is consistent with nonlinear energy balance principles for structures exhibiting amplitude-dependent dissipation. At resonance, the imaginary component of the FRF is inversely proportional to the effective modal damping, which governs irreversible energy loss per cycle. In nonlinear dissipative systems such as degraded timber, effective damping increases with response amplitude due to hysteretic and frictional mechanisms. Increasing the input APSD at the modal frequency, $S_{xx}(f_{n})$, increases the energy delivered to the mode and, consequently, the response amplitude, which in turn elevates the effective damping and suppresses the imaginary FRF component. Under an equivalent linearization conditioned on a fixed excitation level, this interaction leads naturally to an inverse power-law scaling between ImHfn and the input APSD, $S_{xx}(f_{n})$. The observed scaling is therefore interpreted as a physically meaningful consequence of amplitude-dependent energy dissipation rather than a purely empirical correlation.

4. Impulse Range and Reference Input APSD Selection

**Figure 3:**
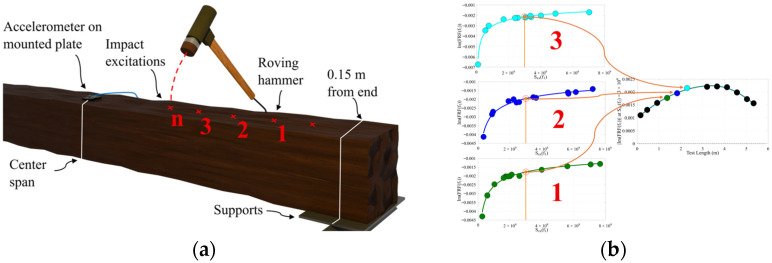
Illustration of (**a**) test configuration with numbered impact locations; (**b**) results diagram for generating the mode shape from multiple impacts and inverse power trends with reference input APSD, $S_{xx}\left(f_{n}\right)$, selection.

The selected impulse range should align with the specific testing objectives. This study adopts a 10 to 20 dB drop in input APSD at the maximum frequency of interest to guide the selection of a reference value of the input APSD for evaluating $Im\left(H\left(f_{n}\right)\right)$, which represents the mode shape amplitude at each location. Figure 3 illustrates the test configuration and post-processing.

The 10–20 dB criterion is not intended to define a unique or globally optimal reference excitation, but rather to identify a stable operating range in which the equivalent linearized response is well-defined, and the inverse power trends are robust. Sensitivity checks indicated that moderate variation within this range produces negligible changes in reconstructed operating shapes, supporting the robustness of the selected reference input APSD. The application of this selection method is outlined in Section 4.4.

### 3.4. Signal Processing Theory

Dynamic testing involves recording the input force from the hammer and the corresponding acceleration response of the structure using an accelerometer, both in the time domain [25]. These time series signals are then transformed into the frequency domain using the FFT. From the FFT results, spectral densities are calculated, specifically, the CPSD between the input and output signals ($S_{xy}$), and the APSD of the input signal ($S_{xx}$). The autopower spectrum is determined by multiplying the input spectrum with its complex conjugate

(1) $$S_{xx}\left(f\right)=X^{*}(f)\cdot X\left(f\right)=(a-ib)(a+ib).$$

The crosspower spectrum is obtained by taking the complex conjugate of the input spectrum and multiplying it by the output spectrum

(2) $$S_{xy}\left(f\right)=X^{*}(f)\cdot Y\left(f\right)=(a-ib)(c+id),$$

where f is the frequency of interest, X(f) is the input spectrum, Y(f) is the output spectrum, and the asterisk (*) indicates complex conjugation. Finally, $S_{xx}$ and $S_{xy}$ are used to generate the FRF, or accelerance, *H*:

(3) $$H\left(f\right)=\frac{S_{xy}(f)}{S_{xx}(f)}.$$

The FRF defines the system’s frequency domain transfer function, relating input force to output response. At resonance, the imaginary component of the FRF reflects the local dissipative response associated with the dominant deformation pattern. In the present study, $Im\left(H\left(f_{n}\right)\right)$ is not interpreted as an invariant linear mode shape amplitude, but rather as an excitation-conditioned operating response metric evaluated at a fixed input power level. The reconstructed shapes therefore represent effective operating deflection shapes (*ODS*) corresponding to the selected reference excitation, rather than global linear normal modes.

The effective *ODS* can be reconstructed by plotting the magnitude of the imaginary component of the FRF at each impact location on the structure. The equation for the effective *ODS* is [26]

(4) $$ODS\left(f\right)=\left[Im\left(H_{1}\left(f\right)\right),Im\left(H_{2}\left(f\right)\right),\ldots ,Im\left(H_{n}\left(f\right)\right)\right],$$

where $H_{n}$ is the FRF at the location of interest. By combining all the imaginary components of the FRF with respect to their location, the effective *ODS* for the structure can be reconstructed.

The SISO roving hammer method utilizes the FRF at each measurement location, relying on the principle of reciprocity. This principle states that the FRF remains the same when the excitation and response locations are swapped. In other words, in an ideal linear-elastic system, impacting at location one and measuring the response at location two yields the same FRF as impacting at location two and measuring at location one. However, in nonlinear systems, variations in impulse magnitude can lead to discrepancies in response, indicating a breakdown in reciprocity as nonlinearity increases. In nonlinear systems, strict global reciprocity may be violated as modal properties evolve with excitation level. However, under a prescribed excitation condition, the system can be interpreted through an equivalent linear representation. The present study therefore assumes conditional reciprocity at a target input power level, enabling the use of a SISO roving hammer approach to extract excitation-conditioned operating mode shapes rather than invariant linear modes.

The theoretical basis for the inverse power relationship between the imaginary component of the FRF and the input APSD that is observed in this study can be supported by considering how observations violate linear systems theory and function consistently with nonlinear energy balance principles. For a structure excited by an impact force, the frequency domain response is

(5) $$Y\left(f\right)=H\left(f\right)\;X\left(f\right),$$

where H(f) is the accelerance frequency response function (FRF) and $X\left(f\right)$ is the force input. For a linear, time-invariant system with viscous damping, the imaginary component of the FRF at resonance $f=f_{n}$ is [14]

(6) ImHfn=12kζ,

which depends only on stiffness k and damping ratio ζ, and is therefore independent of excitation magnitude. In linear theory, varying the input autopower spectral density $S_{xx}(f_{n})$ changes the response energy, but cannot change $Im\{H(f_{n})\}$. Consequently, any systematic dependence of $Im\{H(f_{n})\}$ on $S_{xx}\left(f_{n}\right)$ violates linear modal assumptions.

At resonance, the imaginary component of the accelerance FRF is directly related to energy dissipation. Using the energy definition of equivalent viscous damping [14],

(7) $$\zeta =\frac{E_{D}}{4\pi E},$$

where $E_{D}\;$ is the energy dissipated per cycle and E is the stored modal energy. Generalizing (6), we can say that the imaginary component of the accelerance FRF is proportional to the inverse of the damping ratio

(8) $$Im\{H(f_{n})\}\propto \frac{1}{\zeta }.$$

Thus, reductions in $Im\{H(f_{n})\}$ directly indicate increased effective modal dissipation.

Timber structures commonly exhibit amplitude-dependent dissipation due to hysteresis, micro-slip, crack friction, and support interactions [1]. For such mechanisms, the energy dissipated per cycle scales with response amplitude A as

(9) $$E_{D}(A)\propto A^{2+p},p>0,$$

while the stored modal energy scales as $E(A)\propto A^{2}$. The parameter *p* quantifies how strongly energy dissipation increases with vibration amplitude beyond the quadratic scaling of stored elastic energy; it governs how damping increases with amplitude of vibration. Substituting E and $E_{D}$ into (7) yields an amplitude-dependent equivalent damping ratio,

(10) $$\zeta (A)\propto A^{p}.$$

At resonance, the response amplitude is related to the input APSD by

(11) $$A^{2}\propto \;|H(f_{n}){|}^{2}S_{xx}(f_{n}).$$

Substituting (8) into (11) and considering amplitude-dependent damping (10) yields

(12) $$A^{2}\propto \frac{S_{xx}(f_{n})}{A^{2p}},$$

leading to

(13) $$A\propto [S_{xx}(f_{n}){]}^{1/[2(p+1)]}.$$

Finally, solving for the imaginary component of the accelerance FRF using (8), (10), and (13) yields

(14) $$Im\{H(f_{n})\}\propto [S_{xx}(f_{n}){]}^{-\;\frac{p}{2(p+1)}}.$$

This expression predicts an inverse power-law relationship between the imaginary component of the FRF and the input autopower spectral density at the modal frequency, consistent with the experimentally observed trends. Such behavior cannot be produced by linear modal theory, but arises naturally from nonlinear energy balance when dissipation increases with response amplitude.

### 3.5. Signal Processing and Testing Details

In the testing described here, time series data from the hammer and accelerometer were imported, where the hammer signal was processed by zeroing negative values caused by rebound effects following impact and isolating the impulse region using a low threshold to exclude noise outside the main excitation. An exponential window is generally recommended for responses that do not fully decay [27]. However, in this study, windowing was deemed unnecessary, as the timber beam responses decayed sufficiently within the sampling duration, resulting in minimal leakage. For systems exhibiting slower decay or stronger nonlinearity, windowing may be required. Other window types were tested but introduced undesirable effects, such as amplified noise in the frequency domain. Zero-padding was applied to the time-domain data to effectively interpolate the frequency resolution, increasing the number of frequency bins, thus enhancing the detail in the frequency domain without adding new information. Following this, the FFT was performed, the autopower and crosspower spectra were computed, and the FRF was obtained as the ratio of crosspower to input autopower. This study used a sampling rate of 1600 Hz, 8192 frequency bins, and a spectral resolution (Δ*f*) of 0.0977 Hz.

To assess the effective bandwidth of each test and filter out inadequate impacts, the drop in input APSD should be considered. This study uses the 10 to 20 dB drop guideline [2,20], which states if the drop in input APSD at the maximum frequency of interest is less than 10 dB, the impact may be too strong or hammer tip too stiff, potentially exciting out-of-band modes and losing wanted energy in the modes of interest. If the drop exceeds 20 dB, the impulse is likely too weak or hammer tip too soft, failing to adequately excite the modes of interest. Setting a lower frequency limit was unnecessary, since low-frequency modes are always excited due to the uncontrollable lower bound of the force spectrum in impulse testing. The methodology here incorporates this guideline by evaluating $Im\left(H\left(f_{n}\right)\right)$ at $S_{xx}\left(f_{n}\right)$ from a range of tests in which the maximum frequency of interest falls within a 10 to 20 dB drop in $S_{xx}\left(f\right)$ to adequately excite all modes of interest. While this study chooses the 10 and 20 dB drop values as a guideline, other methods for impact validity are available and the reference input APSD could be chosen differently for other use cases or goals.

### 3.6. Mode Indication

Modal frequencies were identified using the Modal Indicator Function (MIF), with the Component-wise Mode Indicator Function (*CoMIF*) applied to verify mode presence across multiple tests for use within the methodology. The *CoMIF* is defined as [28]

(15) $${CoMIF}_{j}=1-u_{j}\cdot u_{j}^{*}.$$

The *CoMIF* is computed by subtracting the element-wise product of the left singular vectors, $u_{j}$, from a column vector of one, using Singular Value Decomposition (SVD) of the FRF matrix. The asterisk (*) indicates complex conjugation. When *CoMIF* is plotted, the number of curves corresponds to the effective rank of the FRF matrix. Each curve exhibits local minima at the natural frequencies of dominant modes, with the lowest minimum identifying the most dominant mode, which in this study was the first bending mode of the timber specimens. In this study, the *CoMIF* is applied to the ensemble of FRFs generated from valid variable impulse tests at each location to verify consistent modal presence across excitation levels. MIFs become especially important in nonlinear systems, where the natural frequency does not remain constant under varying impact levels. When it comes to damage identification, shifts in natural frequency could be used to infer damage, but the traditional expression for natural frequency depends only on the mass and stiffness of the system, excluding the damping coefficient. As a result, a key variable that could provide insight into structural damage is often overlooked. Damping has been shown to be a more sensitive indicator of structural damage than natural frequency, as it tends to change more significantly in response to such damage [29].

### 3.7. Coherence

The coherence function measures the quality of the frequency domain data by quantifying the linear relationship between input and output signals. It ranges from 0 to 1.0, with 1.0 indicating a perfect linear relationship and 0 representing unrelated signals. In practice, coherence values are typically lower due to noise, system nonlinearity, or multiple excitations affecting the output. Calculating coherence helps assess the accuracy and reliability of the measured data [30].

Traditional coherence methods are calculated using an averaging equation that is not applicable for single impact testing because it results in a coherence of 1.0 for every test [9]. To evaluate nonlinear test validity without averaging, a coherence function developed by Zheng et al. [5], called the Segmentation Method (SM), was specifically designed for individual impact hammer testing without requiring averaging over a dataset. Additionally, coherence will appear poorer in the presence of structural nonlinearity. While the original SM uses two segments, this study uses eight overlapping segments ($N_{s}=8)$ to improve accuracy and reduce sensitivity to noise which can artificially reduce coherence. Overlapping segments increase statistical averaging, reducing the effect of random noise while reinforcing consistent structural signals. However, this comes at the cost of lower frequency resolution, as shorter segments result in wider frequency bins. SM coherence is defined as [5]

(16) $$\gamma^{2}\left(f\right)=\frac{{\bar{S}}_{xy}(f){\bar{S}}_{xy}^{*}(f)}{{\bar{S}}_{xx}(f){\bar{S}}_{yy}(f)},$$

where

(17) $${\bar{S}}_{xx}\left(f\right)=\frac{\sum_{k=1}^{N_{s}}X_{k}^{*}(f)X_{k}(f)}{N_{s}},$$

(18) $${\bar{S}}_{yy}\left(f\right)=\frac{\sum_{k=1}^{N_{s}}Y_{k}^{*}(f)Y_{k}(f)}{N_{s}},$$

and

(19) $${\bar{S}}_{xy}\left(f\right)={\bar{S}}_{yx}^{*}\left(f\right)=\frac{\sum_{k=1}^{N_{s}}X_{k}^{*}(f)Y_{k}(f)}{N_{s}},$$

where the overline denotes averaging over the segments, the asterisk (*) represents complex conjugation, $N_{s}$ is the total number of segments, and $X_{k}\left(f\right)$ and $Y_{k}\left(f\right)$ represent the FFT of the input and output, respectively, during the k-th sampling period. The use of eight overlapping segments represents a practical compromise between statistical stability and frequency resolution; while not formally optimized, this choice balanced adequate resolution for the low-frequency modes of interest with reduced noise.

Coherence can serve as an indicator of whether the input spectrum is adequate for each test. If the input does not sufficiently excite all modes of interest, the power spectrum will “roll-off”, indicating the highest frequency adequately excited [4]. This helps determine whether the impulse was strong enough to capture the structural modes of interest. The coherence plot reveals roll-off where coherence stays near 1.0 within the adequately excited frequency range, then begins to sporadically dip at higher frequencies where excitation is insufficient. This transition is a cue that higher frequencies are no longer being adequately excited, as reflected by increasingly erratic FRF behavior beyond the effective excitation range, indicating the upper limit of adequate excitation. The impulse is determined by swing strength, tip choice of the hammer, mass of the hammer, angle of impact, and contact time, which is often most influenced by the tip choice and test subject material. Importantly, roll-off is not inherently problematic; as long as the mode of interest lies within a portion of the spectrum that is adequately excited, it is acceptable.

### 3.8. Damping

In linear systems, damping is typically modeled as viscous damping and estimated using the logarithmic decrement method, which assumes a constant damping ratio independent of excitation. In contrast, nonlinear systems exhibit damping that varies with input conditions such as impulse magnitude. As impact force increases, damping correspondingly increases, as demonstrated by Blaschke et al. [12] and confirmed in this study. This variation leads to higher damping coefficients and increased damping ratios at larger impulse magnitudes. Nonlinear damping mechanisms often extend beyond viscous effects to include hysteretic damping and Coulomb friction. At low frequencies, hysteretic damping caused by structural anelasticity is typically the dominant energy dissipation mechanism [31]. To accurately capture nonlinear damping effects, this study adopts the instantaneous damping coefficient, a metric recognized as suitable for assessing nonlinear damping behavior [32].

To better understand the physical basis of the FRF and its relation to damping and resonance, it is helpful to consider the system’s dynamics in the frequency domain. The equation of motion (EOM) for a single degree-of-freedom (SDOF) system as a function of angular frequency, ω, is [33]

(20) $$F\left(\omega \right)=U\left(\omega \right)\left(-m\omega^{2}+cj\omega +k\right),$$

where F is the force applied to the system, and j is the imaginary unit. Rearranging the EOM to divide input by output, differentiating the displacement, U,  once to obtain velocity,$\;\dot{U},$ and evaluating the frequency at the resonant frequency, $\omega_{n}$, the damping coefficient of the system, c (N·s/m), is realized. This is detailed in Equations (21) and (22) [33]:

(21) $$\frac{F\left(\omega \right)}{\dot{U}\left(\omega \right)}=\left(c+j(\omega m-\frac{k}{\omega })\right),$$

then

(22) $${\left.\frac{F\left(\omega \right)}{\dot{U}\left(\omega \right)}\right|}_{\omega =\omega_{n}}=c.$$

Acceleration data recorded by the accelerometer was integrated to obtain velocity, enabling the generation of the mechanical impedance (MI) plot, defined as the inverse velocity FRF. Prior to integration, the acceleration signals were band-limited around the modal frequency of interest and zero-mean corrected to mitigate low-frequency drift and numerical bias. The instantaneous damping coefficient was extracted directly at the natural frequency determined by *CoMIF* from the mechanical impedance plot corresponding to the applied impulse level. The damping ratio for each test was then calculated using the natural frequency, the known structural mass, and the instantaneous damping coefficients extracted from the mechanical impedance FRF:

(23) $$\zeta =\frac{c}{2m\omega_{n}}.$$

## 4. Results and Discussion

The test results encompass the application of multiple analytical methods to characterize the behavior of the timber beams and concrete bridge, with interpretations and explanations provided for the observed phenomena. Comparisons will be made with the traditional averaging of FRFs to demonstrate how that approach can yield less accurate mode shapes in the presence of nonlinearity.

### 4.1. Static Load Test Results

**Figure 4:**
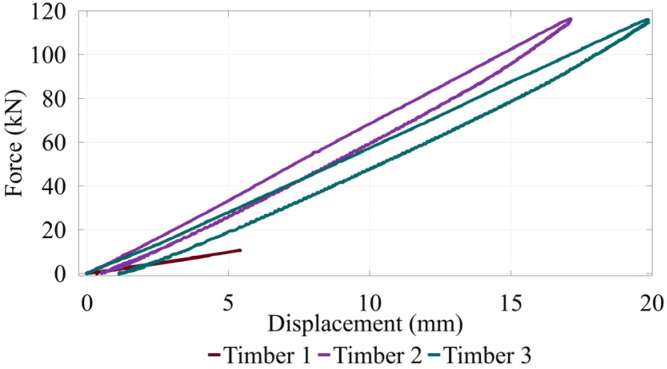
Static load–displacement results for the three timber test subjects.

Static load testing was conducted after dynamic testing was complete to get a baseline for comparing dynamic results. The static load–displacement data for Timbers 1, 2, and 3 in Figure 4 exhibit linear behavior upon loading with limited hysteresis. The stiffness tests give a rough view into the damage in each of the timbers tested. Timbers 2 and 3, with stiffnesses of 6.9 and 5.9 kN/mm, respectively, were much more competent than Timber 1, with a stiffness of 2.0 kN/mm, which was significantly rotted and loaded intentionally less. Nonlinearity was not apparent in static tests even when loading them significantly to the point of audible cracking.

### 4.2. Impact Hammer Test Results

The timber test subjects appeared to exhibit all three types of structural system nonlinearities mentioned by Halvorsen and Brown [6]. Part clearance nonlinearity may have been present from the support conditions. Load-sensitive stiffness nonlinearity was evident, as the timbers appeared to soften (modal frequency decreased) with increasing impulse, and the static stiffness from load–displacement tests decreased under higher loads. Nonlinear damping was evident because damping ratios varied with impulse magnitude, and purely viscous damping was not clearly identifiable from the time series plots.

**Figure 5:**
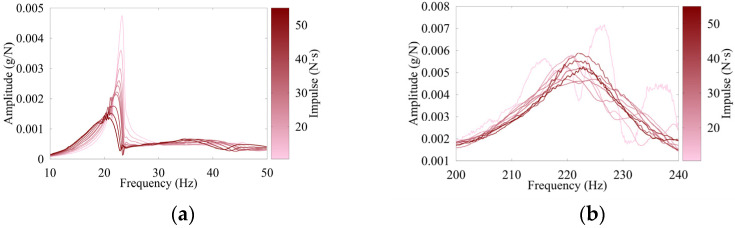
FRF for Timber 3 at 24% of span with varied excitation for (**a**) 1st mode; (**b**) 3rd mode.

The timbers displayed varying degrees of nonlinearity primarily because of their degradation from service. This nonlinearity is evident in Figure 5, which shows accelerance plots for Timber 3, modes 1 and 3. As impulse magnitude increases, both FRF amplitude and natural frequency tend to decrease, highlighting the nonlinear behavior of the timbers, as also observed by Blaschke et al. [12] and shown in Figure 5a. Notably, this nonlinearity causes the FRF results to become increasingly sensitive to impulse variations as the modal frequency decreases. At higher modal frequencies, the grouping of impact tests with varying impulse magnitudes becomes tighter, leading to more consistent FRF results for these modes. However, at lower frequencies, particularly in the first flexural mode, the differences become more pronounced. It is also evident that higher impulse magnitudes improperly excite mode 1, as shown in Figure 5a. This figure illustrates the grouping of FRF magnitudes for both mode 1 and mode 3 and shows that there was greater variance in the lower frequency modes. Similar behavior was observed by Zheng et al. [5], which demonstrated that nonlinearity is more pronounced at lower frequencies, where the differences from varied impulses are more significant. Lighter impacts in the mode 3 plot (Figure 5b) are not smooth as they do not adequately excite mode 3.

### 4.3. Natural Frequencies

The natural frequency was simply determined by the *CoMIF*, where assessing the FRF allowed identification of a natural frequency for each mode. Table 2 presents the natural frequency results for each test subject, obtained from near center-span impacts to isolate the modes of interest. The accuracy of the natural frequency estimates is governed by the spectral resolution of 0.0977 Hz, as described in the methodology.

In general, the natural frequency decreases with increasing impulse magnitude, as larger impulses tend to reduce the dynamic stiffness of the system, leading to a corresponding drop in natural frequency. The typical range of impulses generated were 4 to 61 N·s for the timber beams and 13 to 67 N·s for the concrete bridge. The concrete bridge exhibited more pronounced frequency domain nonlinearities near the supports, suggesting that the impacts were exciting the foundation and thin elastomeric bearing system more effectively than the superstructure, thus diverting the response from the mode of interest.

**Figure 6:**
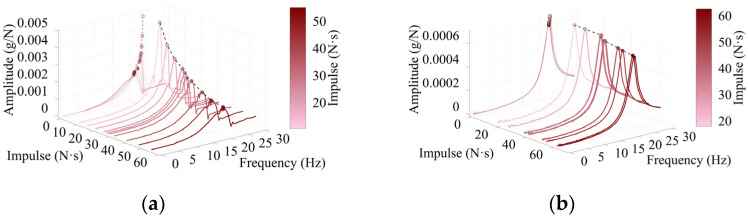
Waterfall plots of frequency and FRF magnitude as a function of impulse with frequency axis 2D projection for (**a**) Timber 3 at 24% of span; (**b**) the concrete bridge at 36% of span.

Figure 6 presents waterfall plots for both the timber beams and the concrete bridge, using varied impacts at the same location in each test.

These plots highlight the variability of FRFs in both frequency and amplitude for nonlinear structures like the timbers. By contrast, the concrete bridge exhibited only minor variations in FRF amplitude and maintained consistent natural frequencies, reflecting its relatively linear behavior. In such linear systems, simple averaging yields reliable results since impulse variation has little effect. However, for the timber beams, FRF inconsistencies caused by varying impulses carry over into mode shape extraction, leading to inaccurate representations. These discrepancies emphasize the sensitivity of mode shape identification to excitation variability, particularly at mode 1, across all timber tests.

**Figure 7:**
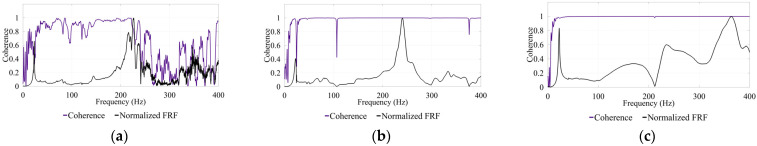
Coherence plots for (**a**) low impulse test on Timber 3 with roll-off around 230 Hz; (**b**) adequate impulse on Timber 2 at 24% of span with clear dips at anti-resonances; (**c**) adequate impulse on concrete bridge.

Examination of the coherence function across multiple tests generally showed reasonable alignment with the excitation content. Most middle-to-high impulse tests had coherence values near 1.0 at modes of interest, since they excited the relevant frequency range effectively. Tests considered less ideal often showed some deviation in coherence (Figure 7a).

A unique feature across all timbers was an anti-resonance immediately after the mode 1 peak, causing poor coherence in that region, as seen in Figure 7b, which is likely due to changes in structural properties or boundary conditions during excitation. The anti-resonance effect was less pronounced in tests with lower impulse magnitudes, indicating that it is impulse-dependent. As a result, the first mode was sometimes clipped, reducing bandwidth and leading to unclean peaks, especially with higher impulses. During processing, coherence helped flag tests insufficient for analysis, which were then excluded. While the nonlinear timbers reached high coherence (Figure 7b), the concrete bridge consistently exhibited coherence values above 0.9 due to minimal nonlinearity in the typical impulse range (Figure 7c).

While amplitude-dependent modal identification frameworks explicitly characterize parameter evolution with response amplitude [34], the objective of VIHIT is to identify effective modal properties at a target excitation level relevant to operational conditions. Conditioning modal parameters on a target input APSD provides compact, repeatable descriptors without necessarily requiring full amplitude-response mapping, which may be impractical or unnecessary for many field applications. However, further exploration of the inverse power trends could serve as a more effective diagnostic tool.

### 4.4. Inverse Power Trend Mode Shape Reconstruction via VIHIT

**Figure 8:**
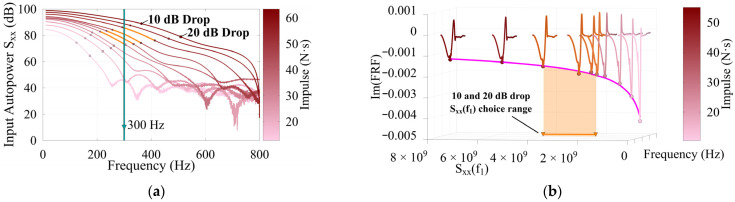
Timber 3 at 24% of span, showing (**a**) 10 and 20 dB drop in $S_{xx}$ with max frequency of interest (300 Hz) for reference $S_{xx}\left(f_{1}\right)$ choice range; (**b**) inverse power trend fitting $Im\left(H\left(f\right)\right)$ versus $S_{xx}\left(f_{1}\right)$, $Im\left(H\left(f_{1}\right)\right)=2.87\cdot {S_{xx}\left(f_{1}\right)}^{-0.34}$, $R^{2}\;=\;0.99$, with reference $S_{xx}\left(f_{1}\right)$ choice range.

This section details the process of reconstruction of the mode shapes using the inverse power trends that result from variable impulse magnitudes. The maximum frequency of interest was set to 300 Hz, which was sufficient to capture modes 1 and 3. At each location, tests were considered valid if the maximum frequency of interest fell within a 10 to 20 dB drop in $S_{xx}\left(f\right)$, indicating that the relevant modes were sufficiently excited and that the $S_{xx}\left(f_{n}\right)$ values were adequate to use as reference. Figure 8a illustrates the typical dB drop from testing based on the input APSD, with the 10 dB and 20 dB drop points clearly marked. Tests in which the maximum frequency of interest falls between these points are highlighted. Figure 8b illustrates the inverse power trend as mentioned in the methodology and reference $S_{xx}\left(f_{n}\right)$ choice range.

Tests conducted at or near the supports mostly yielded lower $R^{2}$ values and more erratic fits to the inverse power trend, likely because impacts there excite the support rather than the beam. The timber specimens’ response at the supports was poorly controlled due to uneven surfaces and perpendicular-to-grain stiffness, further degrading the fit. Points near nodes also showed poor $R^{2}$ due to responses alternating between positive and negative $Im\left(H\left(f_{n}\right)\right)$. At locations or in subjects where the data lie predominantly in the flatter, more linear, portion of the inverse power trend, the mean and inverse power fits are nearly indistinguishable, resulting in low $R^{2}$, while in cases capturing the curved, nonlinear region, data deviate more from the mean and are better described by the inverse power trend, producing much higher $R^{2}$. Still, these locations and subjects generally followed the inverse power trends and overall mode shape trend.

**Figure 9:**
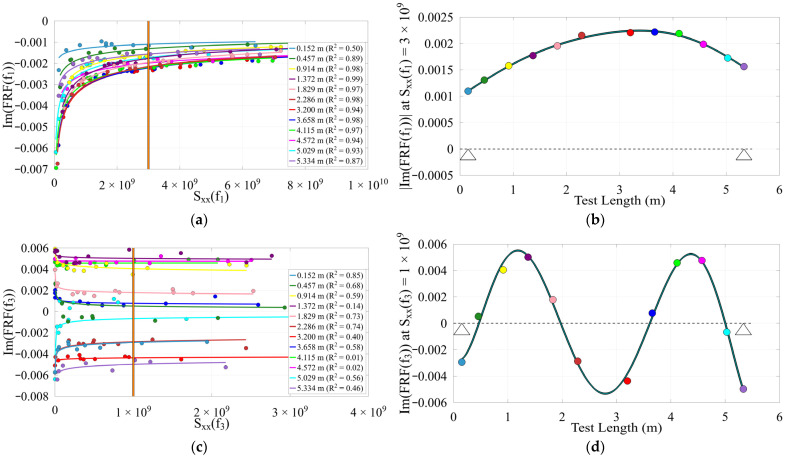
Timber 3 (**a**) 1st mode inverse power trends, $S_{xx}\left(f_{3}\right)$ = $3\;\times \;10^{9}$; (**b**) 1st mode shape (~22 Hz), support locations indicated by triangles; (**c**) 3rd mode inverse power trends, $S_{xx}\left(f_{3}\right)$ = $1\;\times \;10^{9}$; (**d**) 3rd mode shape (~223 Hz), support locations indicated by triangles.

**Figure 10:**
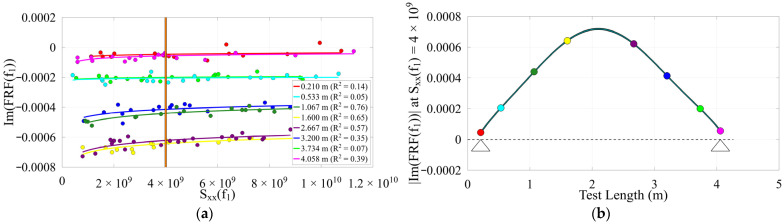
Concrete bridge (**a**) inverse power trends, $S_{xx}\left(f_{1}\right)$ = $4\;\times \;10^{9}$; (**b**) 1st mode shape (~22.5 Hz), support locations indicated by triangles.

**Figure 11:**
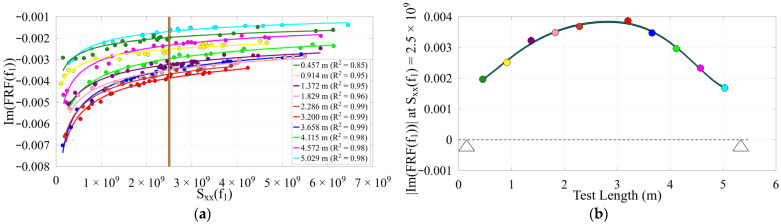
Timber 1 (**a**) inverse power trends, $S_{xx}\left(f_{1}\right)$ = $2.5\;\times \;10^{9}$; (**b**) 1st mode shape (~14.75 Hz), support locations indicated by triangles.

Due to nonlinearity in the timber beams as well as changing section properties along their length, mode shapes deviate from expected patterns, but these deviations may indicate structural damage and can aid in damage identification. This contrasts with the concrete bridge, which behaves more linearly and shows greater consistency in its modal properties. Realistically, slight variance in the mode shape for the concrete bridge may be from the operator’s position during testing, where the added mass effect influences results when that mass is not negligible. Additionally, impact quality can vary due to impact precision and operator fatigue. An example reference $S_{xx}\left(f_{n}\right)$ value from within the overlap of all adequately excited tests at each location was used to evaluate the inverse power trend of $Im\left(H\left(f_{n}\right)\right)$ to produce the mode shape at each location. The reconstructed operating deflection shapes and their inverse power trends are presented in Figure 9, Figure 10 and Figure 11.

As part of the nonlinearity observed, the timbers introduced a somewhat “soft support” similar to modeling with spring supports, due to load-sensitive stiffness nonlinearity such as the rot condition and transverse flexibility of the wood, or part clearance nonlinearity between the beam and supports during variable excitation. The outermost nodes, or locations of zero response, indicating support locations, are not centered at supports but instead are shifted uniquely for each mode as flexural stiffness increases with mode number, while support stiffness stays the same. The natural growth patterns of trees can significantly influence their mode shapes. As tree density and stiffness decrease further from the base, the resulting modal behavior may exhibit skewed shapes. Specifically, the end with lower modal amplitude (Figure 9b) is likely composed of denser, stiffer wood, which corresponds to the base of the tree. When comparing the more linear mode 3 to the nonlinear mode 1 in Timber 3, reciprocity is better maintained, and the shape remains consistent along the entire length (Figure 9d). Due to reduced stiffness from severe rot, Timber 1 exhibited greater modal amplitudes and lower frequencies than the other, stiffer and lighter timbers, and the concrete bridge, with the lower frequencies also influenced by its increased mass.

**Figure 12:**
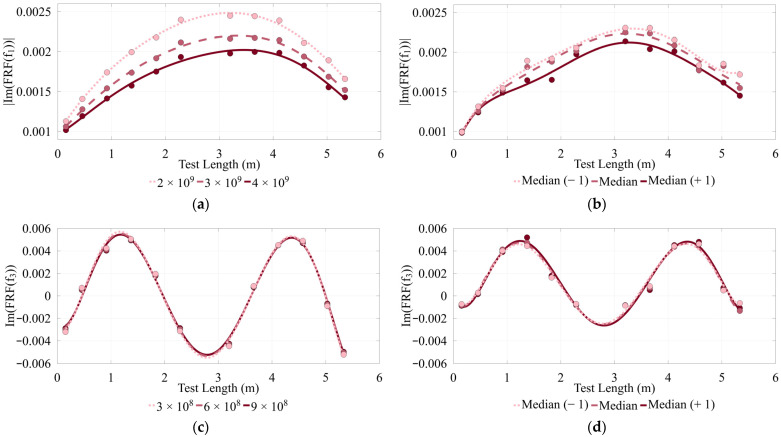
Comparison of mode shapes from inverse power trends (left) and averaging (right) for Timber 3 with (**a**) 1st mode inverse power trend; (**b**) 1st mode average; (**c**) 3rd mode inverse power trend; (**d**) 3rd mode average.

The results in Figure 12 compare mode shapes reconstructed using averaging of $S_{xx}$ and $S_{xy}$ before computing the FRF (Figure 12b,d) versus those obtained using inverse power trends (Figure 12a,c). For averaging, the dataset sorted from least to greatest impulse magnitude was processed using a five-test rolling median window that includes each test and its two neighbors on either side. Shifting this window creates slightly varied subsets, which are averaged to simulate how inconsistent impacts in hammer tests can lead to inconsistent mode shapes in nonlinear structures. The results of this comparison show that using inverse power trends in the VIHIT method produced smoother, more consistent mode shapes (Figure 12a,c), whereas averaging resulted in inconsistent mode shapes (Figure 12b,d). These improved mode shapes are demonstrated in the reduction in higher order terms in polynomial regression along with small but improved coefficients of determination. MAC values also demonstrate improvements, with a negligible difference between methods for mode 1 (MAC value of 0.9978) but more significant difference for mode 3 (MAC value of 0.7573). Off-diagonal terms were also evaluated, yielding 0.0192 (Mode 1 averaging vs. Mode 3 inverse power trend) and 0.3077 (Mode 1 inverse power trend vs. Mode 3 averaging). Off-diagonal terms for the inverse power trend auto-MAC are 0.0226 and for the averaging method auto-MAC are 0.2961, indicating greater orthogonality for the mode shapes identified by VIHIT than by using averaging.

### 4.5. Estimated Damping Ratios

**Figure 13:**
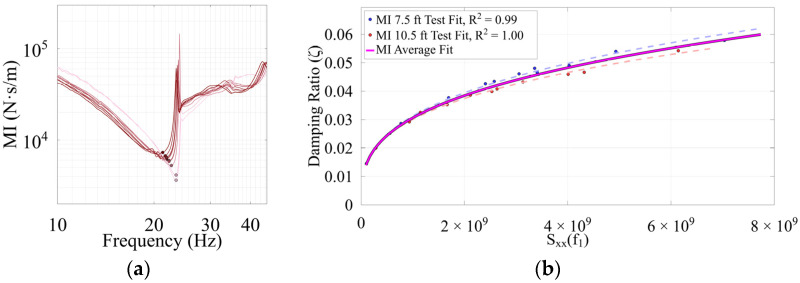
Timber 3 damping ratio extraction with (**a**) damping coefficient from the mechanical impedance FRF plot near mid-span (2.3 m); (**b**) example damping ratio results, power trend fit.

To estimate damping, damping coefficients were derived from mechanical impedance FRF plots from tests near modal antinodes, with the damping ratio estimations calculated accordingly. Ideally, testing directly at the antinode would be preferred, but this requires finer spatial resolution and precise knowledge of the antinode’s location, which may not be possible for all structures. An example of damping coefficient acquisition from the mechanical impedance plot and the corresponding damping results using Equation (23) for Timber 3 is shown in Figure 13.

The damping ratio tends to increase with increasing impulse. This is typically true; however, certain modes sometimes show no sign of a damping trend, or a smaller damping ratio with higher impulse [12]. This behavior may result from nonlinear effects where damping becomes amplitude-dependent due to internal friction. Higher impulse magnitudes cause larger displacements, which can amplify these nonlinear responses and alter the apparent damping in the time series. In practice, the same $S_{xx}\left(f_{1}\right)$ chosen to generate the mode shape could also be used to estimate damping simultaneously. Interestingly, the damping ratio for Timber 1 decreased significantly in its severely rotted state compared to the other sound timbers. This may suggest that advanced rot transformed the timber into a dense but weak homogeneous material, likely reducing hysteretic damping effects. In contrast, Timber 2 and 3 likely exhibited higher damping due to increased internal friction from early-stage decay and damage, including microcracking. Damping ratios are detailed in Table 3.

The primary objective of this study is to improve consistency in impact hammer-based identification under nonlinear conditions rather than to benchmark absolute accuracy against shaker-based or controlled excitation methods. While independent reference testing would be valuable, such comparisons are beyond the scope of the present experimental configuration and are identified as an important direction for future work.

## 5. What Can Go Wrong?

Hammer testing is fraught with potential error, much of which can be reduced with careful conduct of testing. Overload and saturation are limitations specific to the accelerometer that can degrade test quality, but understanding and accounting for them, as discussed by Avitabile [35] and Brown et al. [25], can significantly improve the reliability of impact hammer modal testing. Double impacts, caused by hammer rebound followed by an unintended second strike, appear as a secondary peak in the time series and introduce oscillations in the FFT and APSD. These effects degrade data reliability and should be identified and removed before processing. Operator fatigue from using heavier hammers can negatively impact test quality, and inconsistent impacts due to misalignment, hammer size, or poor operator positioning introduce measurement errors. To improve consistency according to the method proposed here, it is recommended to perform at least 10 trials per test location. Additionally, poor accelerometer mounting can lead to vibration at the mount, introducing noise seen as choppy or erratic fluctuations in the frequency domain. Discarding poor trials and identifying problems like low signal-to-noise ratio contribute to more accurate and reliable results.

## 6. Future Recommendations

The methodology proposed here is simpler than using controlled impact systems or additional equipment, offering a cost-effective solution that leverages common modal testing tools and portable data acquisition. Although gathering sufficient data can be demanding, improvements like filtering low-quality tests and using the reference $S_{xx}\left(f_{n}\right)$ to guide and optimize attempted hammer impulse can streamline the process, reducing the number of impacts needed to reliably develop inverse power trends within the target decibel-drop range. Real-time analysis could allow operators to monitor impact precision and receive instant coherence and FRF feedback, enabling them to generate appropriate impulses immediately to account for the entire trend and improve repeatability.

In impact hammer modal analysis, low-frequency data is often contaminated by sensor limitations, such as poor near-DC response and drift. Employing sensors with improved low-frequency performance can significantly reduce noise and enhance the accuracy of modal parameter estimation such as dynamic stiffness for damage identification with the VIHIT method proposed here.

With a structured testing program, this method could produce valuable data for structures that are typically difficult to instrument and assess, such as rural road bridges. Building a data history would enable more accurate assessment of structural health and support proactive maintenance decisions and better-informed load postings. Although this project did not begin with a well-characterized, undamaged beam, future work should incorporate one and introduce controlled damage such as material cutouts, hollow sections, pockets of rot [15], or progressive flexural damage [16]. Impact testing at various locations could then reveal how nonlinear modal responses shift near the modified areas, providing clearer indications of typical damage behavior. Ideally, future work could compare natural deterioration, such as uncontrolled rot in timber beams, with changes in modal properties and damage detection. Performing consistent inverse power trend analysis at each location would help quantify how nonlinear structural behavior changes with material removal. However, since damage such as wood rot combined with timber splits can unpredictably alter capacity and dynamics, local damage identification may not always be effective. In such cases, tracking global modal changes may be more reliable. Modeling may also be necessary to apply certain damage identification techniques effectively.

As frequency increases, the effect of impulse variability diminishes, reflecting each mode’s unique response to the energy input. Quantifying how linearity improves with higher frequency modes could help improve understanding of structural conditions, especially when evaluating changes in higher order modes over time.

## 7. Conclusions

This paper presents the development and validation of the Variable Impulse Hammer Impact Test (VIHIT), a new experimental approach for identifying modal parameters in structures with impulse-dependent responses. The method aims to improve the reliability of impact hammer modal analysis for lower frequency, nonlinear systems using a simple SISO roving hammer and fixed accelerometer setup. Traditional SISO testing relies on averaging and is often unsuitable for nonlinear structures due to impulse-dependent responses. VIHIT addresses this by applying a range of impact magnitudes at each location, computing FRFs, and analyzing trends in FRF amplitude and damping as functions of input APSD at modal frequencies identified via MIFs. By extracting inverse power trends between the input APSD at a modal frequency and the imaginary component of the FRF, VIHIT simulates a consistent impulse level to determine corresponding response values, enabling more accurate reconstruction of mode shapes and standardized excitation-conditioned modal properties. Damping is estimated from similar trends in mechanical impedance plots and linked to the reconstructed mode shapes using the same reference input APSD, thereby accounting for nonlinear response behavior. Since modal properties such as frequency, damping, and stiffness vary with impulse magnitude in nonlinear structures, VIHIT provides a practical means to characterize these actual variations by reducing uncertainty in the impulse level. Its simple setup and minimal equipment requirements make the method highly accessible and cost-effective compared to existing modal analysis techniques. These advantages make VIHIT a viable, reproducible method to improve damage detection, structural health monitoring, and structural assessment, especially for large structures like bridges with damaged or degraded members, where the hammer’s size and impulse capacity enhance both efficiency and portability. Moreover, this methodology is applicable to smaller impact hammers and mechanical systems, broadening its use in various mechanical engineering applications.

## Figures and Tables

**Table 1:** Timber cross-sectional properties, dimensions, and mass.

Test	*b* (mm)	*d* (mm)	*I* (mm^4^)	Span (m)	Length (m)	Mass (kg)
Timber 1 ^1^	343	397	1.79 × $10^{9}$	5.2	5.5	577
Timber 2 ^1^	343	397	1.79 × $10^{9}$	5.2	5.5	513
Timber 3 ^1^	343	397	1.79 × $10^{9}$	5.2	5.5	488
Concrete Bridge ^2^	1194	254	1.63 × $10^{9}$	3.9	4.3	3731

^1^ Values are as accurate as measured, with considerable uncertainty in moment of inertia due to rot, damage, warping, and cracking in beams. ^2^ Mass was assumed via calculations with normal weight reinforced concrete (2400 kg/m^3^).

**Table 2:** Mode 1 natural frequencies for test subjects recorded with impacts near center-span.

Subject	Mode 1 Natural Frequency (Hz)	Increase	Mode 3 Natural Frequency (Hz)	Increase
Timber 1	14.6–15.2	4.7%	132.3–134.9	1.96%
Timber 2	20.4–23.4	14.8%	240.8–243.1	0.96%
Timber 3	20.1–23.7	17.9%	220.0–224.9	2.23%
Concrete Bridge	22.3–22.8	2.2%	N/A	N/A

**Table 3:** Damping ratio estimate.

Subject	$\mathrm{Damping}\;\mathrm{Ratio}\;\mathrm{at}\;S_{xx}\left(f_{1}\right)$ = $3\;\times \;10^{9}$	$\mathrm{Damping}\;\mathrm{Ratio}\;\mathrm{at}\;S_{xx}\left(f_{3}\right)$ = $1\;\times \;10^{9}$
Timber 1	0.0222	0.0251
Timber 2	0.0348	0.0227
Timber 3	0.0439	0.0255
Concrete Bridge	0.0208	N/A

## Data Availability

The raw data supporting the conclusions of this article will be made available by the authors on request.
